# Epidemiology of skin changes in endangered Southern Resident killer whales (*Orcinus orca*)

**DOI:** 10.1371/journal.pone.0286551

**Published:** 2023-06-28

**Authors:** Joseph K. Gaydos, Judy St. Leger, Stephen Raverty, Hendrik Nollens, Martin Haulena, Eric J. Ward, Candice K. Emmons, M. Bradley Hanson, Ken Balcomb, Dave Ellifrit, Michael N. Weiss, Deborah Giles

**Affiliations:** 1 The SeaDoc Society, Karen C. Drayer Wildlife Health Center - Orcas Island Office, UC Davis School of Veterinary Medicine, Eastsound, Washington, United States of America; 2 SeaWorld Parks and Entertainment, San Diego, California, United States of America; 3 Animal Health Center, Ministry of Agriculture, Abbotsford, British Columbia, Canada; 4 Vancouver Aquarium, Vancouver, British Columbia, Canada; 5 Conservation Biology Division, Northwest Fisheries Science Center, National Marine Fisheries Service, National Oceanic and Atmospheric Administration, Seattle, Washington, United States of America; 6 Center for Whale Research, Friday Harbor, Washington, United States of America; MARE – Marine and Environmental Sciences Centre, PORTUGAL

## Abstract

Photographic identification catalogs of individual killer whales (*Orcinus orca*) over time provide a tool for remote health assessment. We retrospectively examined digital photographs of Southern Resident killer whales in the Salish Sea to characterize skin changes and to determine if they could be an indicator of individual, pod, or population health. Using photographs collected from 2004 through 2016 from 18,697 individual whale sightings, we identified six lesions (cephalopod, erosions, gray patches, gray targets, orange on gray, and pinpoint black discoloration). Of 141 whales that were alive at some point during the study, 99% had photographic evidence of skin lesions. Using a multivariate model including age, sex, pod, and matriline across time, the point prevalence of the two most prevalent lesions, gray patches and gray targets, varied between pods and between years and showed small differences between stage classes. Despite minor differences, we document a strong increase in point prevalence of both lesion types in all three pods from 2004 through 2016. The health significance of this is not clear, but the possible relationship between these lesions and decreasing body condition and immunocompetence in an endangered, non-recovering population is a concern. Understanding the etiology and pathogenesis of these lesions is important to better understand the health significance of these skin changes that are increasing in prevalence.

## Introduction

Skin disease has been used as a remotely sensed indicator of health in many cetacean species, including bottlenose dolphins (*Tursiops truncatus*) [1,2], common minke whales (*Balaenoptera acutorostrata*) [3], Guiana dolphins (*Sotalia guianensis*) [4], North Atlantic right whales (*Eubalaena* glacialis) [5,6], and others [7–9].

Understanding skin changes, including skin disease, in Southern Resident killer whales (*Orcinus orca*), a small, endangered population of fish-eating salmon specialists [10], could provide insight into morbidity or help predict mortality [6,9]. This population ranges through coastal and inland waters from Chatham Strait in southeastern Alaska (USA) to Monterey Bay, California (USA) and is structured socially into three pods (J, K, and L). The population reached its smallest size (n = 71) in 1975, increased into the mid-1990’s, then decreased again before being listed as Endangered in Canada and the United States (Center for Whale Research Census data). Despite recovery efforts, the population is not moving towards recovery targets [11] and fewer than 75 animals remain. To identify threats and improve recovery, causes of mortality have been reviewed [12] and a body condition scoring system has been developed for individual animals, pods, and the population [13]. However, little is known about the role disease, including skin disease, could play in limiting population recovery.

Since 1976, the Center for Whale Research has conducted Southern Resident killer whale photographic identification surveys in the Salish Sea to capture clear images of every whale. While the larger goal has been to census and track the population status and demographics, the photographs have also been valuable for other purposes such as assessing social affiliations [14]. When evaluating these high-resolution photographs, biologists have noted transient and occasionally persistent abnormal skin changes, but these have never been characterized or tracked over time or by individual animal.

Numerous infectious agents can cause skin lesions in cetaceans. Examples of reported bacterial etiologies include *Aeromonas hydrophila* [15], *Corynebacterium* spp. [16], *Dermatophilus*-like organisms [17], *Erysipelothrix rhusiopathiae* [18], *Mycobacterium* spp. [19,20], *Pseudomonas aeruginosa* [21], and *Vibrio* spp. [16]. Pathogenic fungi known to cause cutaneous lesions in cetaceans include *Candida* spp. [22,23], *Fusarium* spp. [19,24], *Lacazia loboi* [25–27], which is now recognized as *Paracoccidioides ceti* [28], as well as mucoralean fungi [29,30]. Cetacean poxviruses are known to cause “tattoo” skin lesions and focal skin discoloration in cetaceans [31]. Other viral etiologies of cetacean skin disease include calicivirus [32], alpha- and gamma-herpesviruses [33,34], and papillomaviruses [1,7,35].

Anthropogenic, interspecific, and intraspecific trauma are common non-infectious causes of skin lesions in some cetacean species [36–38]. Further, environmental factors, such as solar ultraviolet radiation [39], water temperature [40] and low salinity [41,42], are known to cause vesiculobullous lesions and dermatitis as well. Finally, ectoparasites such as ciliates (*Kyaroikeus cetarius*) [43], copepods (*Pennella balaenopterae*; [3,44], nematodes [36,45], cookie cutter sharks (*Isistius* spp.) [3,46], and several species of sea lamprey (*Entosphenus tridentata* and *Petromyzon marinus*) [3,47–49] also cause skin injuries in cetaceans. Congenital disorders including anomalously white and partial lack of epidermal pigmentation also can alter the appearance of the epidermis in some cetacean species [38,50].

The delineation between infectious and non-infectious causes of skin disease is not always clear, and often dermatitis in cetaceans can be caused by trauma and secondary infection [17,44,51]. Systemic immunosuppression associated with viral infection also can predispose cetaceans to dermatitis [43]. More direct associations between viral infections and secondary microbial invaders also can occur [24]. Additionally, environmental factors can interact with infectious agents to cause skin disease. For example, freshwater skin disease can predispose animals to secondary bacterial, fungal, and algal colonization, proliferation, and deeper tissue invasion [41].

In some cases, skin lesions in cetaceans are associated with impending mortality. For example, Hamilton and Marx [6] showed that “swath” lesions that are likely associated with fishing gear entanglement are often associated with fatal outcomes in North Atlantic right whales. Van Bressem et al. [9] noted expansive annular lesions in a Chilean dolphin (*Cephalorhynchus eutropia*) calf that merged and involved between 30–40% of the visual body surface before the calf was no longer sighted and presumed dead.

In killer whales specifically, skin changes can occur from multiple causes. These include anthropogenic trauma [52,53] and conspecific trauma [54–56]. Also, freshwater skin disease can cause a moderate to severe, variably extensive erosive and ulcerative dermatitis with superficial and deep epidermal fissures in killer whales [12]. Ectoparasite-associated skin lesions in killer whales include round to oval full-thickness lesions consistent with cookiecutter shark bite wounds [12,46], superficial round and serrated skin lesions from the sea lamprey *P*. *marinus* [57], grayish marks that may be a sequela to remora (*Echaenidae*) attachment [58], and dermatitis caused by invasive ciliates [43]. Unclassified poxvirus has been detected in “tattoo” or “ring” skin lesions [33] in killer whales and papilloma virus intranuclear virus-like particles were described in hyperplastic epithelial lesions [59]. Epidermal lesions of unknown etiology also have been reported [9,38].

Compared to other cetacean species, killer whales have a relatively high occurrence of anomalously pigmented individuals [60], which is hypothesized to occur because killer whale populations are often relatively small with low genetic diversity [61]. Chediak-Higashi syndrome, an autosomal recessive disorder, was diagnosed in a live-captured female transient killer whale [62]. In terrestrial animals and humans, this condition renders individuals highly susceptible to infection; however, it is unknown if all anomalously colored killer whales have this congenital disease.

Photographs taken of surfacing free-ranging cetaceans have been used to classify skin disorders and study their epidemiology worldwide [3,4,9,63–68]. While this relatively simple and minimally invasive approach does not permit identification of specific etiologies, it can be used to better understand the importance of skin lesions as a measure of health, especially when capture-release health assessments are not possible [69,70].

The goal of this study was to identify and characterize skin changes, their occurrence, and their association with mortality in endangered Southern Resident killer whales using high-resolution digital photographs taken for photo identification purposes. While we did not try to identify the etiologies of skin disease, we did work to quantify changes in the presence of skin lesions temporally (season and year), as well as by age, sex, pod, and relationship to mortality.

## Materials and methods

### Data

We evaluated digital photographs of surfacing Southern Resident killer whales taken by the Center for Whale Research (CWR). Specifically, photographs collected for identification purposes focused on the left and right saddle patch and dorsal fin [71]. All photographs were from the bi-national Salish Sea and were collected under United States and Canadian federal permits (NMFS 15569; DFO SARA 272, respectively) as previously described [14]. To mitigate bias, the retrospective analysis only used data collected by high-resolution digital cameras, which began in 2004. Only a small number of experienced biologists photographed animals over the duration of the study and all images were of known individual Southern Resident killer whales. Every in-focus, clear photograph taken between 2004–2016 was evaluated for skin abnormalities. Four veterinarians, two veterinary pathologists specializing in marine mammals and two veterinarians with expertise in cetacean clinical medicine, collectively reviewed all images of skin lesions on high-definition screens. Distinct lesions were identified and differentiated by typical pathologic qualifiers: lesion type (e.g., abscess, depressed, nodular, papillary, proliferative, raised, ulcerated, scarred, vesicular), color (e.g., dark, pale, gray, red, white), and distribution (e.g., coalescing, diffuse, focal, multifocal, patchy, pinpoint). A name and morphologic description were given for each distinct lesion type and every lesion present in an image was recorded by lesion type, date, and individual animal.

Skin disease data were joined with the CWR sightings and demographic data. Age and sex were converted to stage classes, following the conventions of Ward et al. [72]: calves (0–1 years old), juveniles (1–9 years old), young reproductive females (ages 10–42), young males (ages 10–21), old post-reproductive females (43+), and older males (22+).

### Summarizing lesion prevalence

For each type of skin lesion, we summarized the number of lesion occurrences and absences from 19,807 killer whale sightings (combinations of unique animals and days). If an animal was seen during an encounter but a lesion was not identified in any of the photographs taken of the animal that day, we assumed a lesion was absent. Aggregate summary statistics for each lesion type were generated at the population and stage class level. We considered additional summaries by social group and season, but no meaningful differences were found between groups or seasons. Data are concentrated during the summer months when Southern Resident killer whales frequent this area, and most photographs are taken.

### Modeling lesion prevalence

To understand patterns in killer whale skin lesion prevalence, we developed a series of statistical models, using lesion occurrence (0/1) as the nominal response variable. While we modeled the probability of individual lesion occurrence, these estimates can also be thought of as point prevalence in a population where occurrence is assumed to vary independently by individual. Point prevalence (the proportion of animals with a particular lesion at a particular timepoint) is calculated as the number of cases in the Southern Resident killer whale population on a certain day divided by the number of animals in the population on that day. When occurrences are independent across individuals, point prevalence will be equal to the probability of occurrence (individuals with lesions being drawn from a binomial distribution with constant probability). If there are factors related to family or social groups that contribute to point prevalence, other methods that incorporate social grouping would be expected to produce different estimates.

As some lesion types were rare, we focused most of our modeling efforts on the three most frequently observed lesion types: gray patches, gray targets, and pinpoint black lesions. Models for each of these three lesions (binomial family, logit link) were constructed using a generalized additive modeling (GAM) framework, using the R package mgcv [73,74]. GAMs provide a flexible approach for modeling variation, including allowing for smooth relationships to vary similarly by population groups [75]. To understand how the occurrence of these lesions changed seasonally, across years, and between segments of the population (by stage class, and pod), we developed separate GAMs for each lesion that included seasonal smooths by pod, annual smooths by pod, and both pod and stage-specific fixed effects. As each of the three pods has slightly different distributions in a year, seasonal and annual smooths were unique to each pod (letting each have a different pattern over time) but constrained to have the same smoothness. The inclusion of pod and stage fixed effects only affects the intercept, allowing overall lesion occurrence to vary by group. Additional models were also considered in our initial exploration, including models with shared smooths across pods or random intercepts varying by individual; as these terms were not found to improve predictive performance (quantified with AIC, cross-validation), we did not consider them further.

Hypotheses were limited for less common skin lesions (cephalopod, erosions, and orange on gray) and incidental dermatologic findings (calf epithelial sloughing and fetal folds) because of the sparsity of the data. We fit simple models to these data, however, to evaluate evidence for annual and seasonal trends. Annual trends for less common skin lesions and incidental dermatologic findings were evaluated by fitting binomial generalized linear models (GLMs) with an intercept and year trend in R [74]. Similarly, seasonal trends were estimated by fitting generalized additive models (GAMs [75]) with an intercept and smooth over month (cyclic cubic regression spline).

### Ethics

Institutional Review Board Approval was not required for this study as it did not include any interaction or intervention with human subjects or any access to identifiable private information.

## Results

### Skin condition occurrence

From 2004 through 2016, the three most prevalent skin lesions were gray patches, gray targets, and pinpoint black discoloration (Table 1). Cephalopod lesions, erosions, and orange on gray were rare (Table 1). One or more of the three most prevalent skin lesions were noted in 99% (140 of 141) of Southern Resident killer whales alive at some point during the study period. From one to four distinct lesions were noted on a single animal. Additionally, we identified fetal folds and calf epithelial sloughing; two normal conditions that are observed in the epidermis of perinates and calves (Tables 1 and 2). The only whale without skin lesions was K41, who only lived for 4 months.

**Table 1:** Prevalence of skin changes in Southern resident killer whales across all pods and age classes (2004–2016), except for age-specific lesions. Calf epithelial sloughing and fetal fold lesions are only summarized for animals < 2 years of age, and orange on gray lesions only for calves and juveniles.

Skin Change	Days Absent	Days Present	Total	%
Calf epithelial sloughing	217	51	268	19.0%
Cephalopod	5,414	46	5,460	0.8%
Erosions	5,399	61	5,460	1.1%
Fetal folds	244	24	268	9.0%
Gray patches	105	5,355	5,460	98.1%
Gray targets	731	4,729	5,460	86.6%
Orange on gray	5,448	12	5,460	0.2%
Pinpoint black	3,280	2,180	5,460	39.9%

**Table 2:** Skin change occurrence in Southern Resident killer whales by stage class (Ward et al., 2013) [72].

Skin Change	Calf	Juvenile	Young Female	Old Female	Young Male	Old Male	Total
Calf epithelial sloughing	51	0	0	0	0	0	51
Cephalopod	0	10	26	1	7	2	46
Erosions	4	6	48	0	3	0	61
Fetal folds	24	0	0	0	0	0	24
Gray patches	198	1,233	1,951	484	1,115	374	5,355
Gray targets	130	1,075	1,876	237	1,068	343	4,729
Orange on gray	10	2	0	0	0	0	12
Pinpoint black	18	438	837	67	615	205	2,180

### Skin condition descriptions

#### Gray patches

**Fig 1:**
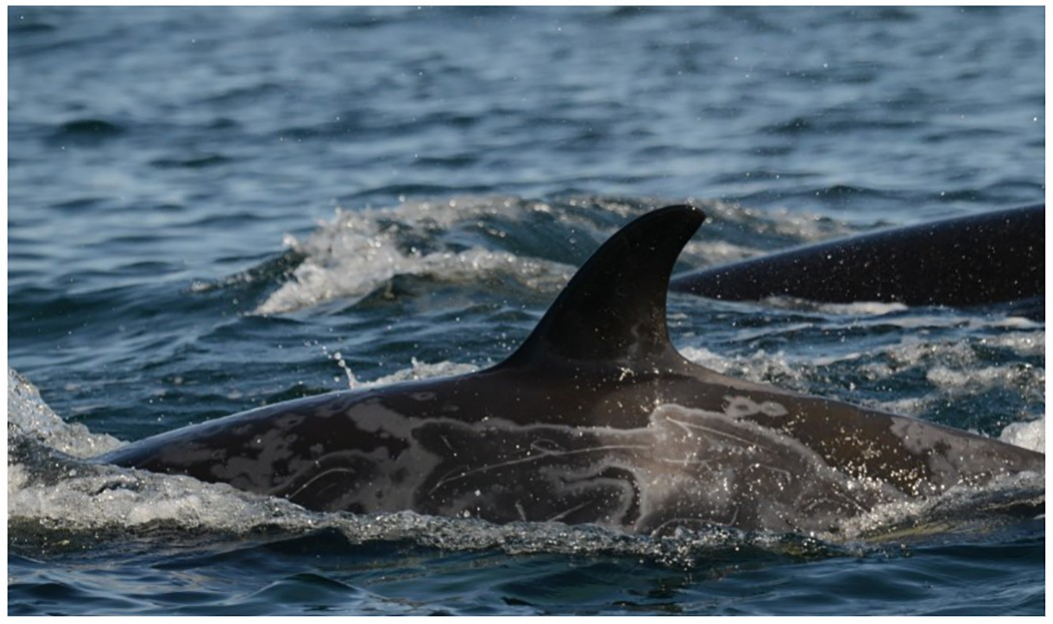
Variably extensive and occasionally coalescing gray patches on Southern Resident killer whale L121 (June 13, 2015).

**Fig 2:**
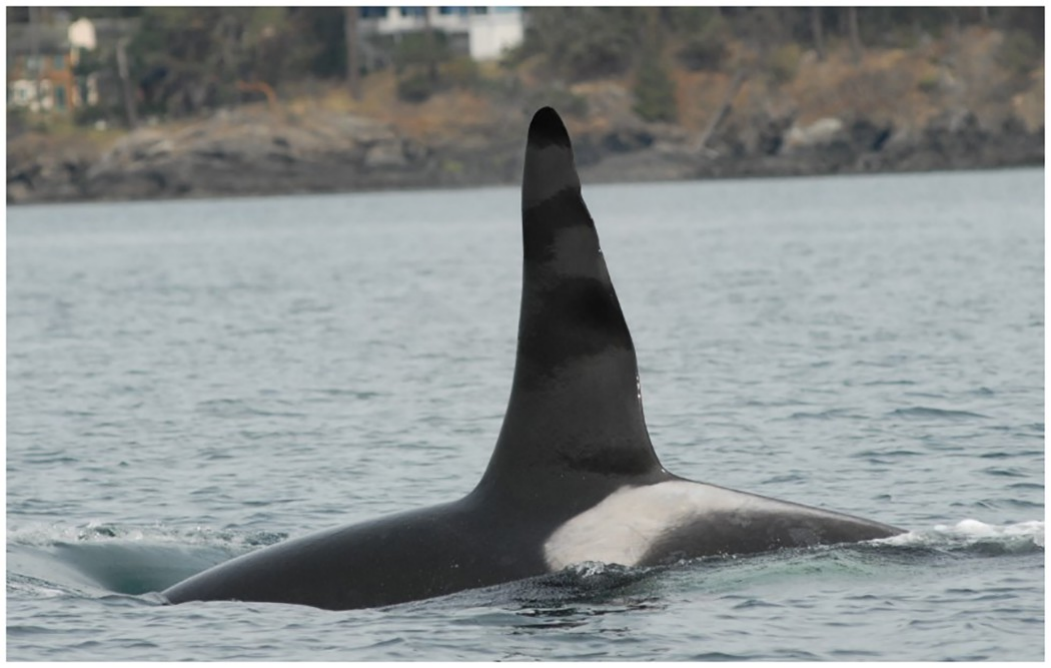
Multiple gray patches on the left lateral aspect of the dorsal fin of Southern Resident killer whale J1 (April 1, 2008).

Occurrences of multifocal and/or variably extensive discrete gray discoloration of the epidermis (Figs 1 and 2) were designated as gray patches and were the most noted lesion, observed in 5,357 of 19,807 sightings (27%) and found in all life stage classes (Tables 1 and 2). They ranged from focally disseminated to variably extensive and were observed randomly throughout regions of exposed epidermis. They could be poorly circumscribed or discrete and had serpiginous to curvilinear margins. The lesions did not have a predilection to any specific anatomic region and varied in size and shape within different affected anatomic regions. Depending on the animal and timing of the photo series, the lesions were dynamic and expanded and contracted in size over time. We hypothesized gray patches could be epidermal edema (spongiosis) or possibly, hyperplasia. Occasionally serial images over time showed gray patches were associated with, and delineated, rake marks and gray targets.

#### Gray targets

**Fig 3:**
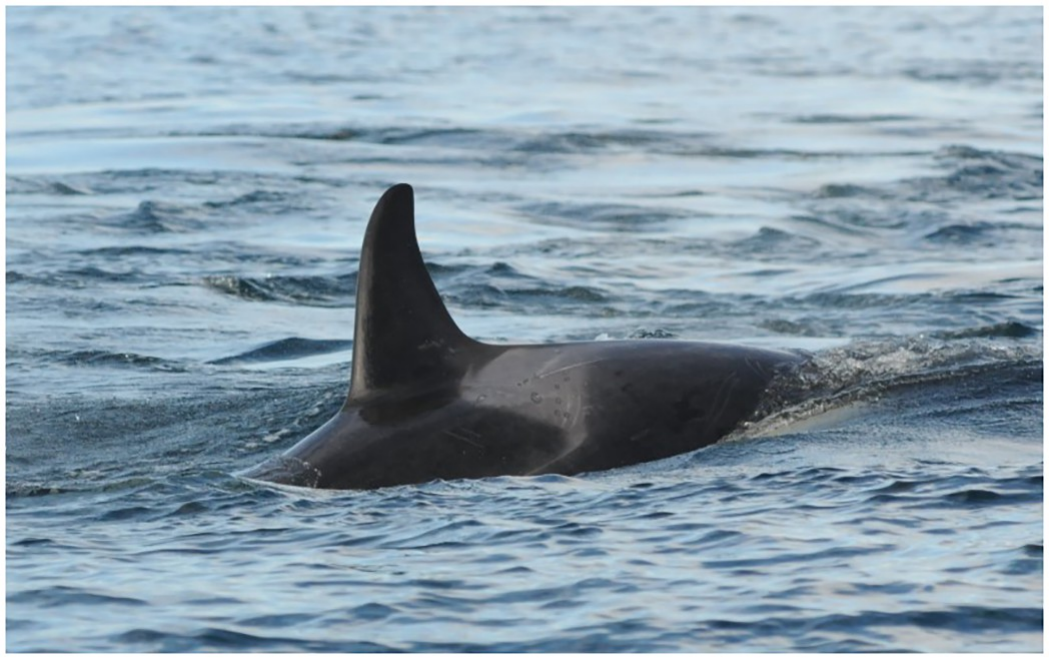
Southern Resident killer whale L118 showing gray targets in saddle patch (October 18, 2014).

Gray targets were the second most detected skin lesion, noted in 24% of whale sightings (4,731 of 19,680). Like gray patches, these lesions were observed in a variety of anatomic sites and in all life stages, including a neonate with fetal folds (Tables 1 and 2). These concentric, alternating two-toned lesions were well delineated and multifocal to coalescing (Fig 3). Margins were usually darker than the center and occasionally a time series of photographs showed progression to, or development of supervening gray patches.

#### Pinpoint black discoloration

**Fig 4:**
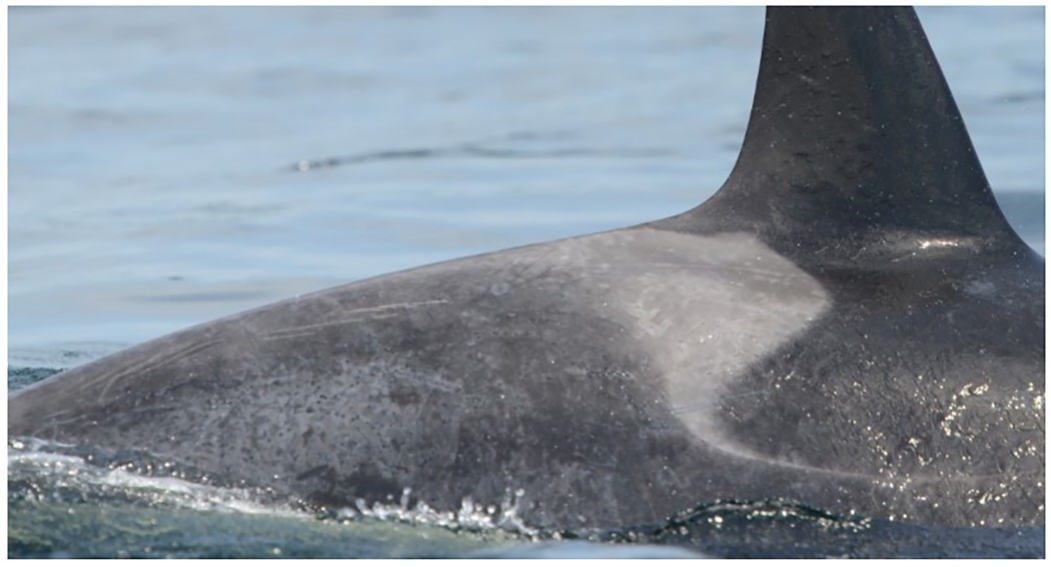
Multifocal pinpoint pitted black discolorations circumscribed by gray patches on the right saddle patch and caudally on Southern Resident whale L109 (August 18, 2014). Many are clearly associated with healing rake marks.

Multifocal punctate black erosions, termed pinpoint black discoloration, were seen in all life stages, and documented in 2,180 of 16,965 sightings (11.4%; Tables 1 and 2). These lesions were often within rake marks (Fig 4). For example, on June 27, 2008, fresh rake marks were noted on the left flank of L85, a 17-year-old male. Fourteen days later (July 11), pinpoint black discolorations were visible in the healing rake marks. The rake marks had progressed into linear gray patches when the animal was photographed 28 days later (August 8), at which time the pinpoint black marks had resolved.

#### Erosions

**Fig 5:**
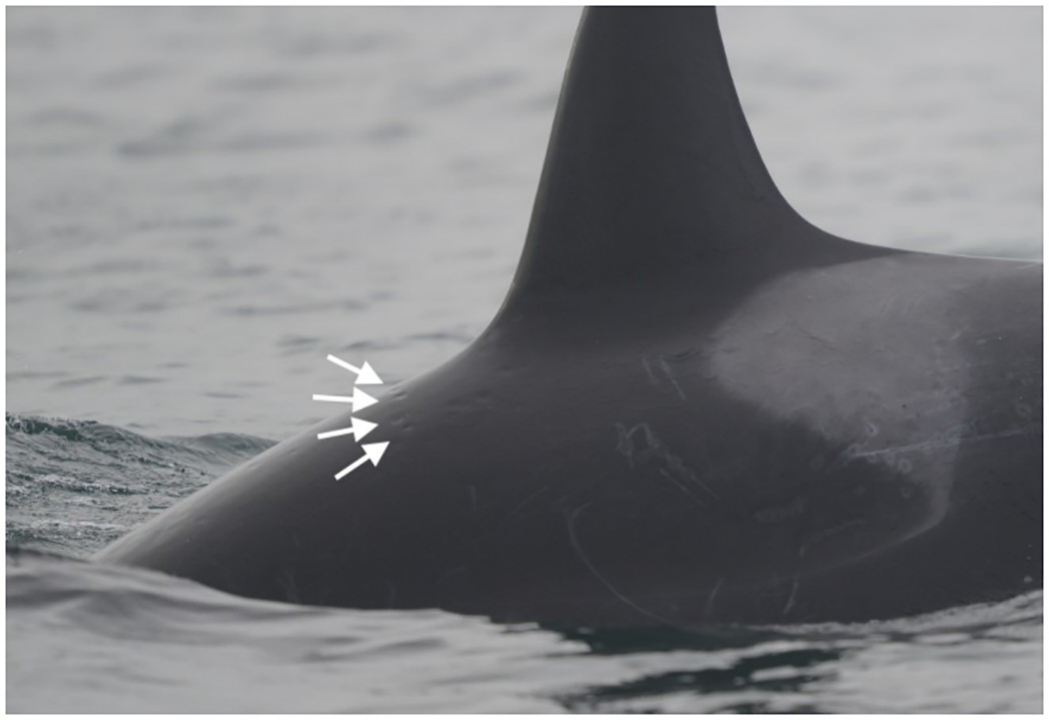
Well circumscribed depressed areas of skin attributed to loss of superficial layers of epidermis just cranial to the dorsal fin of Southern Resident killer whale L116 (November 26, 2014).

Well circumscribed depressions with superficial loss of skin layers and no discoloration were defined as erosions (Fig 5). These lesions were rare, observed in only 49 of 18,697 sightings (Table 1) and only in calves, juveniles, and young males and females, predominantly young females (Table 2).

#### Cephalopod lesions

**Fig 6:**
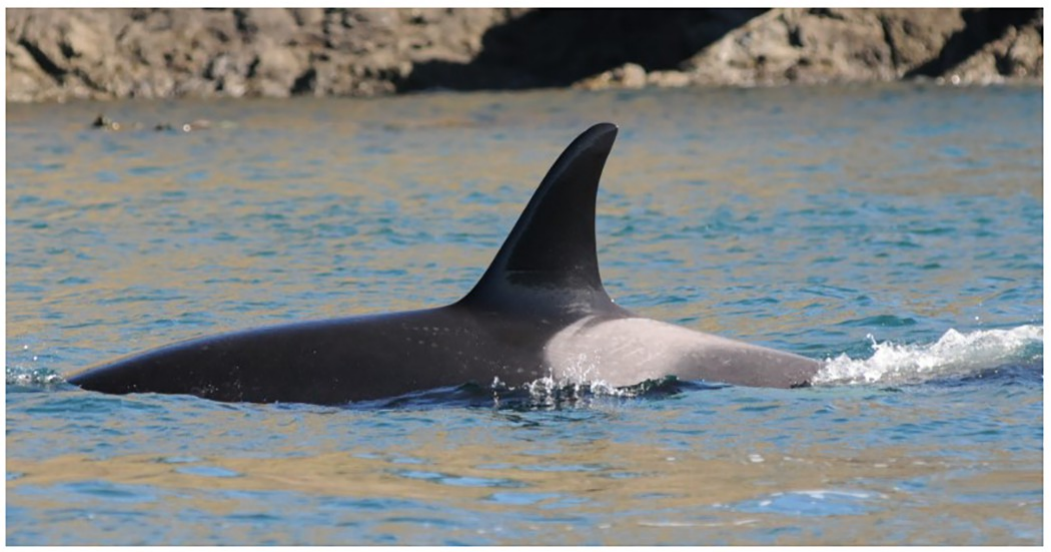
Multiple linear, regularly spaced, circular lesions cranial to, within, and extending behind the saddle patch on the left lateral flank of Southern Resident killer whale K036 (October 3, 2010).

Intermittent equidistant circular lesions less than 5 cm in diameter occurred in linear or curvilinear fenestrated tapering rows that were progressively smaller distally were rare, occurring in 30 individuals and accounting for 0.2% of sightings (Table 1). These lesions were visible on the dorsal and lateral aspect of animals as far rostral as the level of the mandibular ramus and immediately caudal to the blow hole and as far distal as the tail stock. As the lesions were longer than rake marks and often appeared to taper distally like the suction cups on the tentacle of a cephalopod, we called these cephalopod lesions (Fig 6). These were seen in all life stages except calves (Table 2), were seen in almost every year of the study, and on five occasions were noted on two different animals in a population on the same day. In 10 of 30 individuals, lesions were noted on more than one occasion.

#### Orange on gray

**Fig 7:**
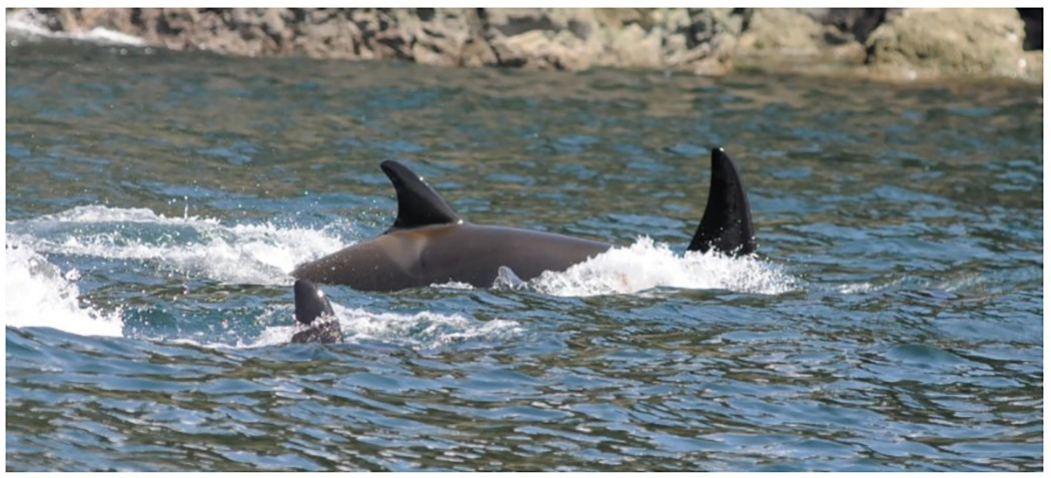
Orange on gray lesion at margin of dorsal fin and saddle patch on the right side of Southern Resident killer whale neonate J52 (May 1, 2015). Fetal folds are also visible.

Orange on gray lesions were noted in nine animals and were characterized by an orange hue, often without well delineated margins, partially or completely covering the gray color of the saddle patch or found just above the saddle patch at the insertion of the dorsal fin (Fig 7). The lesion was noted 12 times in nine animals, all calves and juveniles. For eight of these animals, the lesion was noted during their first year of life. In one of those animals, it appeared to have resolved, then re-occurred at 2 years of age. For the ninth animal, it was not noted the first year of life, but was seen once in year two. The lesion was seen concurrent with fetal folds in 20% (n = 3) of the 15 animals photographed with fetal folds during the study. For one, the lesion was visible when fetal folds were present (Fig 7), then again 16 days later, at which time the fetal folds were no longer visible.

### Modeling point prevalence of skin conditions

**Fig 8:**
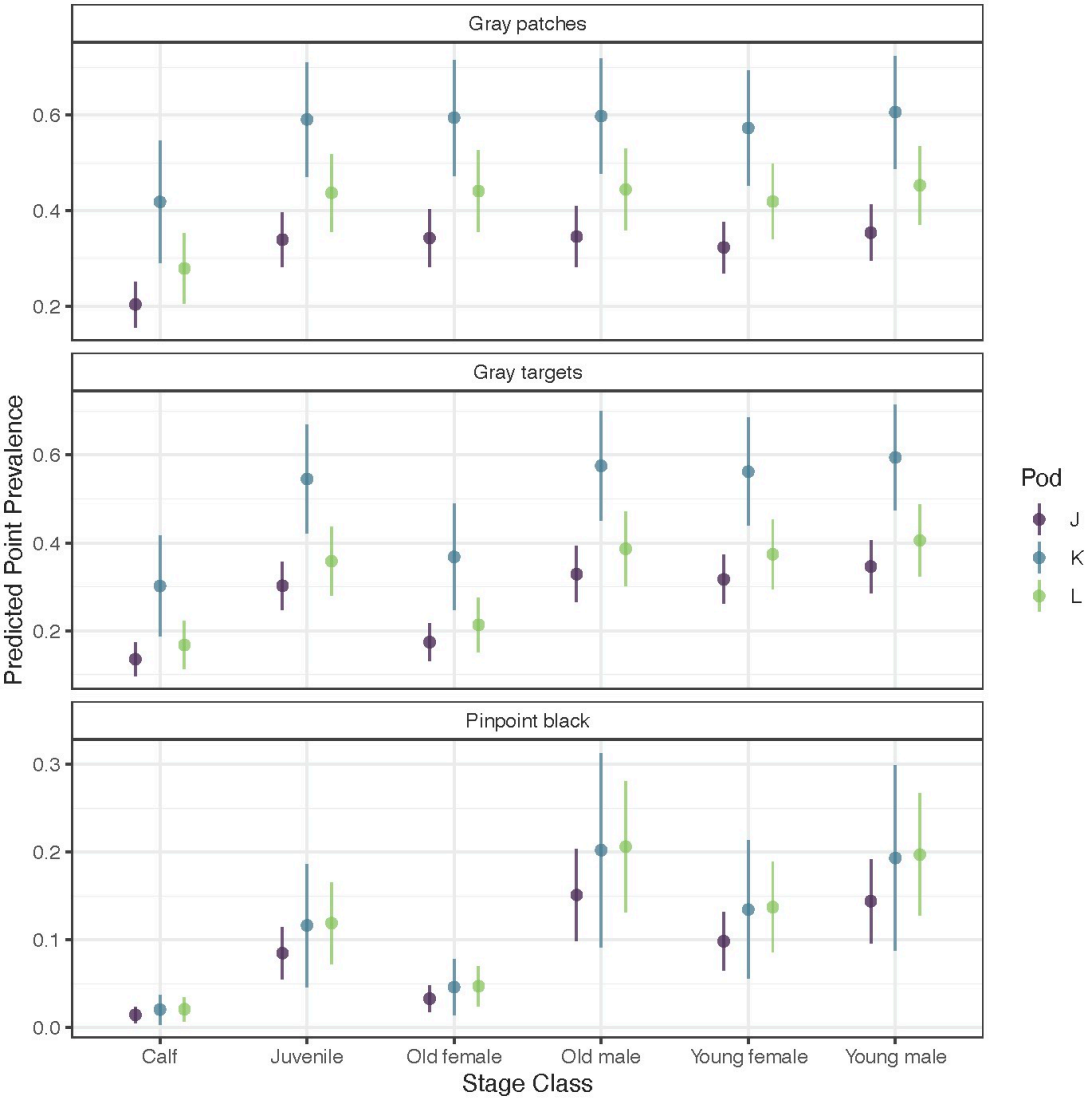
Predicted point prevalence (probability of occurrence) of gray patches, gray targets, and pinpoint black discolorations for each pod (J, K, and L) by stage class (stages are assumed to have the same trend, and only differ by an intercept in link space). To look at the effect of pod and stage, other covariates are held constant, showing the predictions at the 200^th^ calendar day (July 19) in 2016, a mid-way point. Point estimates represent predicted means, and vertical lines represent 2 standard errors.

Modeling occurrence of gray patches, gray targets, and pinpoint black discoloration revealed a high degree of correlation between gray patches and targets. There were distinct differences in predicted point prevalence of gray patches and gray targets between killer whale pods and stage classes (Fig 8). Predicted point prevalence for gray patches and gray targets was highest for K pod and lowest for J pod, with only minor differences noted between J and L pods (overlapping standard errors). By stage class, calves had the lowest point prevalence of both gray patches, gray targets, and pinpoint black discolorations. All three pods had similar prevalence of pinpoint black lesions, with J pod having slightly lower prevalence, but overlapping standard errors (Fig 8). By stage class, calves and old females had lower predicted point prevalence compared to other stage classes (Fig 8). This stage class difference also can be seen for gray targets, but only for J- and L-pod, and not for K-pod (Fig 8).

**Fig 9:**
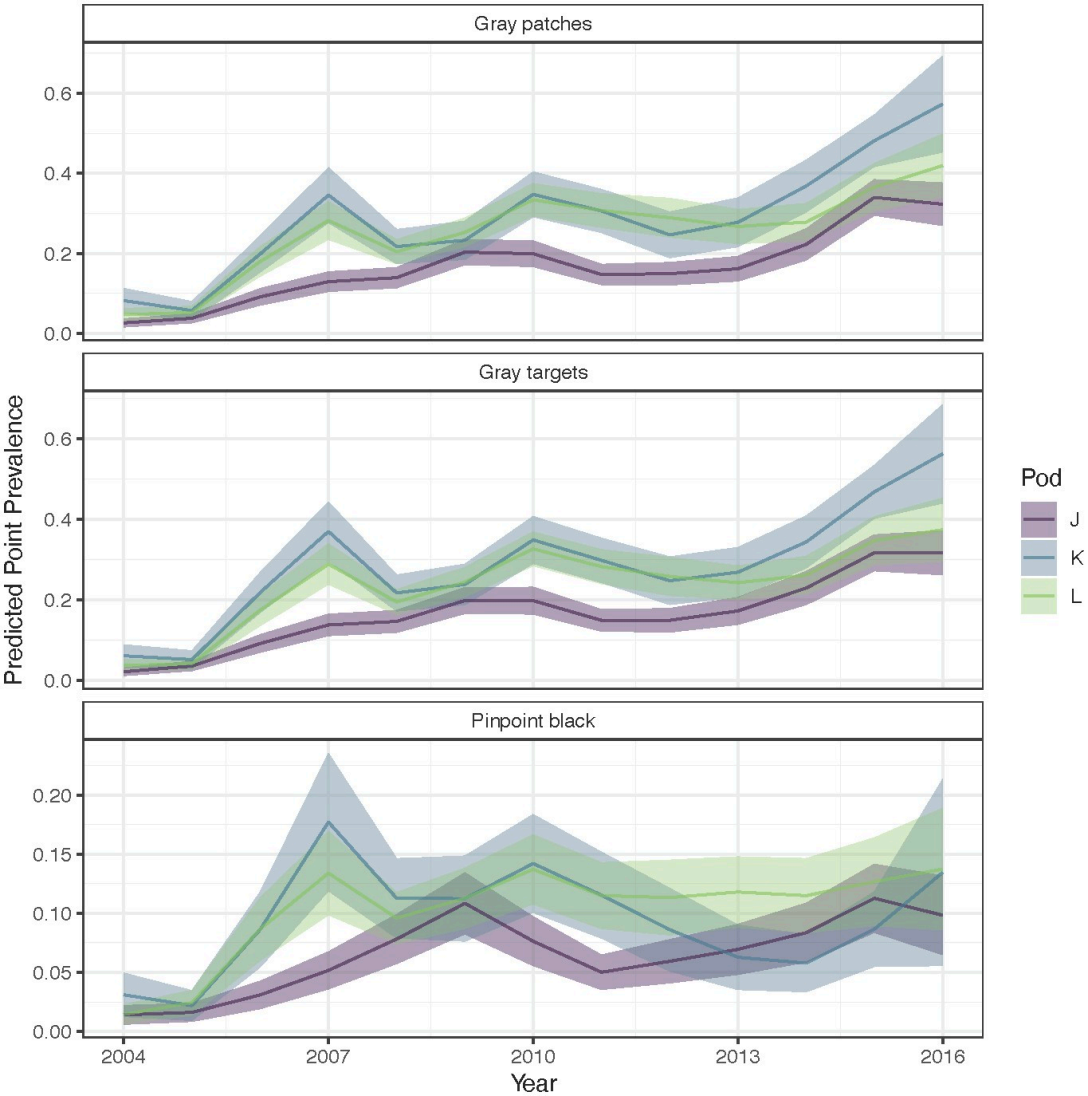
Estimated annual variation in predicted skin lesion point prevalence by pod. To look at the effect of pod and year, we hold other covariates constant and show young female killer whale stage class (11–42 years old). Means are shown with a solid line, and ribbons represent 2 standard errors.

Over the study period, point prevalence of gray patches and gray targets increased for all three pods (Fig 9). These did not increase monotonically. For example, K and L pods had lower point prevalence from 2007–2009 and again between 2011–2014, while J pod had increasing point prevalence from 2007–2009 and slightly increasing point prevalence from 2011–2014. Overall increases in point prevalence of gray patches and gray targets across years appear to be highly correlated between K and L pods, with a different estimated overall pattern for J pod (Fig 9). As seen with stage classes (Fig 8), over time predicted point prevalence for gray patches and targets is highest for K pod and lowest for J pod, with L pod overlapping both.

While the point prevalence of pinpoint black lesions increased between 2004–2007, their overall occurrence appears to be relatively flat from 2007–2016 (Fig 9). Correlations between K and L pods can be seen early in the time series, but since 2008, all pods appear to have diverged to a unique pattern with no consistent trend.

**Fig 10:**
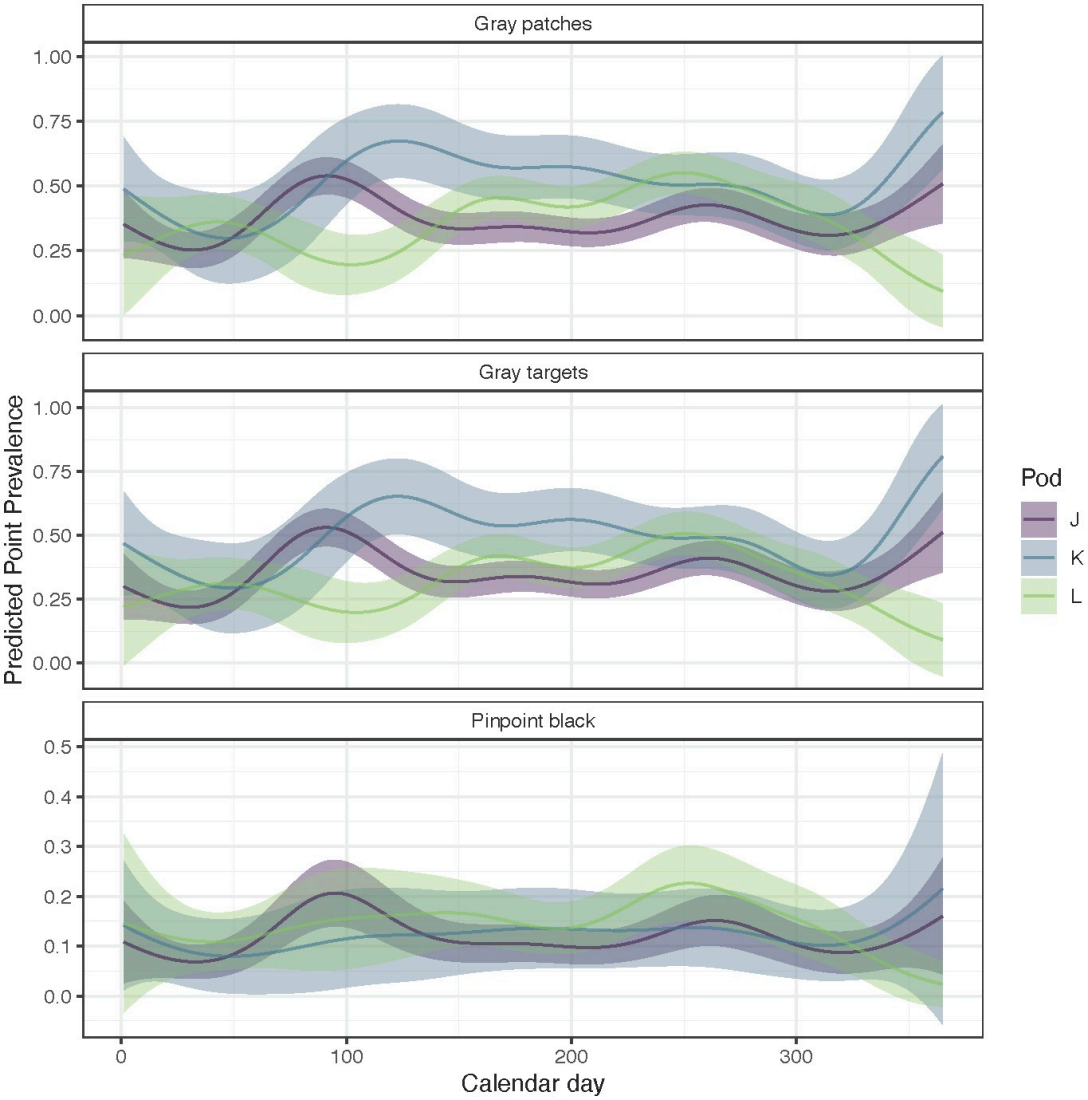
Estimated seasonal trends in skin lesion occurrence, by pod. To look at the effect of pod and season (calendar day), we hold other covariates constant and show young female killer whale stage class (11–42 years old). Means are shown with a solid line, and ribbons represent 2 standard errors.

When examining seasonal changes in lesion occurrence we can make predictions for the entire calendar year (Fig 10). However, we focused our inference on the May–October period when whale sightings were highest (S1 Fig). Estimates of lesion occurrence in this time interval suggest that there are contrasting patterns between K and L pods. Point prevalence of gray patches and gray targets decrease for K pod but increase for L pod (Fig 10). The predicted occurrence of gray patches and gray targets for J pod is highest in spring and relatively constant over summer months (Fig 10). As with annual changes, we found less support for clear seasonal patterns or differences between pods in the occurrence of pinpoint black lesions. Trends for J and L pod appeared like those of other lesions; however, the trend for K pod was estimated to be flat or slightly increasing (Fig 10).

We did not find significant linear (yearly) trends for any of the less prevalent conditions (cephalopod, erosions, orange on gray) or for incidental dermatologic findings. Except for calf epithelial sloughing, the seasonal effects of less common skin conditions were not significant. The estimated seasonal trend for calf epithelial sloughing was significant (p < 2e^-16^), with the occurrence estimated to be highest in spring and lowest in late summer months (S2 Fig).

### Incidental dermatologic findings

#### Fetal folds

**Fig 11:**
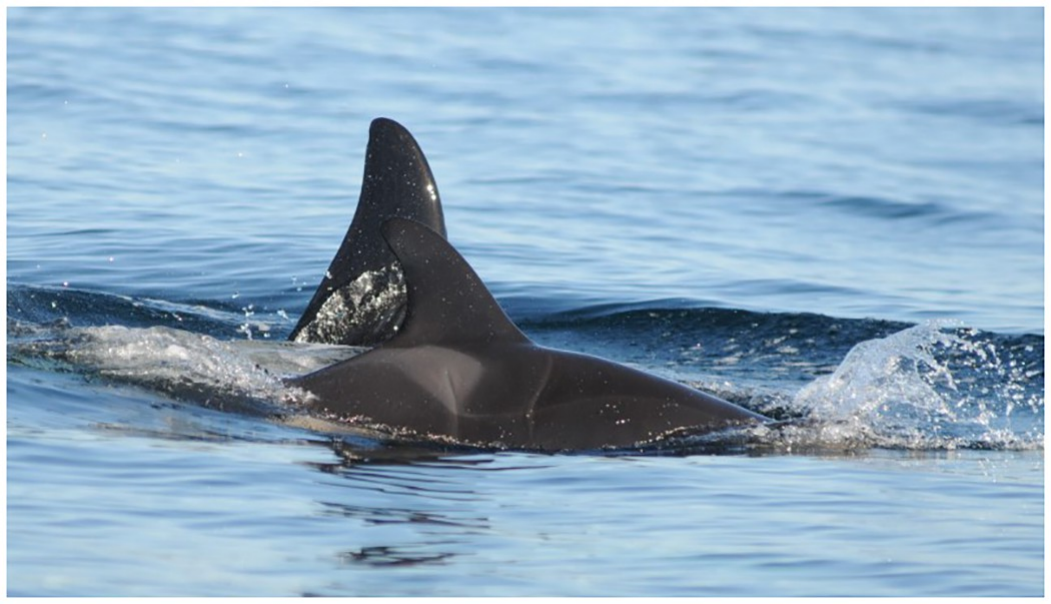
Two fetal folds visible on the right flank of Southern Resident calf L120 (September 6, 2014).

*In utero*, killer whale neonates curve laterally to conserve space. The fetal abdomen is directed towards the maternal head, and the fetus’ head and tail are directed towards the maternal tail [76]. After birth and as the fetal body straightens, light colored bands and shallow vertical grooves are visible along the skin of the lateral abdomen on what was the concave side of the fetus *in utero*. We identified 24 observations of fetal folds in 15 animals during the study (Fig 11). For some animals, folds were visible on both sides of the body. Sequential sightings of fetal folds from four animals revealed that actual folds or lightly pigmented bands at the location of past folds were visible for up to 55 days after first detection.

#### Calf epithelial sloughing

**Fig 12:**
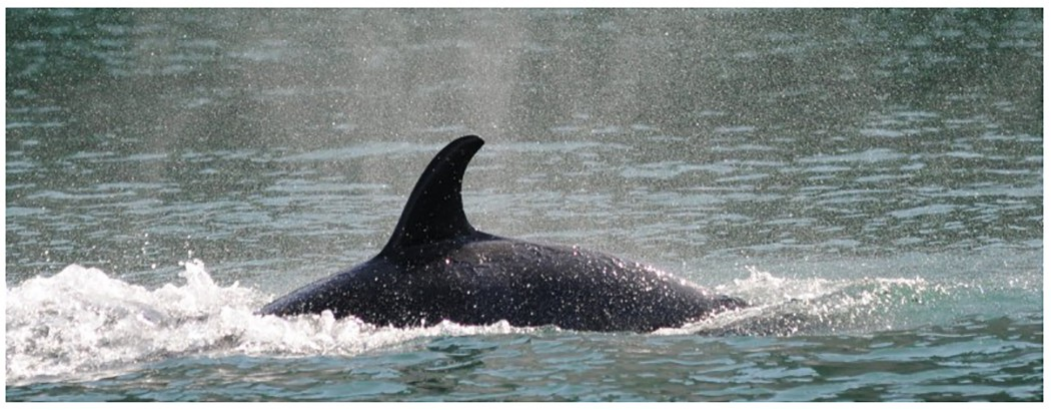
Focal to patchy, epithelial hyperplasia with detachment of the superficial layers, termed calf epithelial sloughing, seen in Southern Resident killer whale calf J53 (February 25, 2016).

Focal to patchy, epithelial hyperplasia and possible hyperkeratosis with epithelial separation and sloughing and occasional cleft formation (Fig 12) were seen on 51 occasions in 25 calves. These observations were consistent with postnatal ecdysis [77], as documented in other cetaceans. We defined this condition as calf epithelial sloughing. For the 14 calves in which the condition was detected in sequential sightings, it persisted for up to 91 days. In nine animals, fetal folds were observed prior to calf epithelial sloughing and subsequently, the two conditions were seen simultaneously.

## Discussion

Examination of photographs of Southern Resident killer whales in the Salish Sea identified six actual skin lesions (cephalopod lesions, erosions, gray patches, gray targets, orange on gray, and pinpoint black lesions). We also identified two normally occurring skin conditions (fetal folds and calf epithelial sloughing). Of the six examples of disease, three were frequently observed. An annular increase in point prevalence of the two most common lesions, gray patches and gray targets, is concerning.

While some skin pathogens like cetacean poxviruses [33], *Erysipelothrix rhusiopathiae* [18], and mucoralean fungi [30] can cause mortality in cetaceans, including killer whales, our modeling did not suggest that any of the six lesions we identified are correlated with mortality.

As expected, retrospective analysis of high-quality digital photographs taken to identify killer whales represents an accessible way to examine skin disease in this species. Multiple studies (e.g., [37,65,78,79]) have shown that photo identification data provided an efficient and cost-effective approach to document the occurrence and nature of skin lesions in other free-ranging cetacean species. Photographs collected opportunistically for identification purposes do have limitations, however. Free-ranging cetaceans are not sighted consistently (over days or even seasons), creating irregularly spaced and, often, large gaps between animal sightings. Some groups of Southern resident killer whale individuals tend to be sighted more frequently than others (e.g., J pod occurs much more frequently in the inland waters of the Salish Sea). In this study, such gaps prevented us from definitively understanding skin lesion progression, duration, and possible resolution. Also, photographs taken for identification purposes generally show only the portion of the animal that was exposed at the surface. This prevented us from detailing the distribution of lesions over the entire body and could have caused us to miss lesions occurring on less visible parts of the body. Potential causes for false negative (Type II-like) errors, where a lesion was present but not identified, include lesions not visible to the photographer (e.g., occurring on an area of the body below the water surface or positioned away from researchers), water cascading over the skin, or poor lighting that produced glare or another anomaly that could have obscured a lesion. Finally, while we understood this at the onset of the study (and hence not a goal of our investigation), photographs alone do not permit diagnosis of etiologies underlying skin disease. The categories are descriptive rather than diagnostic. We realize that similar etiologies may result in different presentations.

### Gray patches and gray targets

The presence of gray patches and gray targets was highly correlated. The two lesion types were most prevalent and may represent two distinct pathogenic mechanisms or represent two components on a continuum of a single disease process. Point prevalence of these two lesions varied between pods and between years and showed small differences between stage classes. There was little difference in lesion point prevalence by season. Most strikingly, there was a strong increase in lesion prevalence from 2004 through 2016 in all three pods (J, K, and L; Fig 9). We considered that increasing trends over time could be an artifact of sampling or bias, but this was not supported by the data. Hypothetically, researchers could have taken more photos of lesions over time as they became aware that the lesions existed; however, this does not seem to be the case. The overall increasing annual trend in point prevalence of gray patch and gray targets is not perfectly linear and does not monotonically change as one would expect if driven by observer bias. Both gray patches and gray targets showed increases in point prevalence up to about 2010, then declined from 2012–2014, only to increase again sharply for an overall increasing trend over the study period. Photographers were highly consistent over the duration of the study, and it is unlikely that observer bias would increase steadily, then drop, and then rise again while also varying by pod. Instead, we hypothesize that variation in predicted point prevalence of gray patches and targets, including the increasing trend over time, is real and likely related to one or more unknown external drivers. As these skin lesions are considered an expression or manifestation of disease process and seem to be increasing in all three pods, understanding their etiology and pathogenesis and relationship to external drivers is important to determine if it is related to lack of population recovery.

The distinct annual increases in prevalence of two correlated skin lesions in endangered Southern Resident killer whales may not be unique to this population or geographic region. Van Bressem et al. [9] reported an exponential increase in reports of skin disorders in cetaceans worldwide. They hypothesized this increase was related to a causal link with markedly deteriorating coastal environments, climate change, and mounting levels of solar ultraviolet radiation that could exacerbate cutaneous diseases by inducing DNA damage in the epidermis. We do not have data to support this hypothesis in the case of Southern Resident killer whales.

While we are unable to comment on the etiology of gray patches and gray targets, their increasing occurrence in Southern Resident killer whales could be related to other stressors previously associated with skin lesions in cetaceans like water temperature and salinity. A large study [65] comparing prevalence of skin disease in ten coastal bottlenose dolphin populations in multiple oceans did not find a relationship between skin disease and contaminant levels. Instead, they found a significant linear relationship between the occurrence of skin disease and several oceanographic variables. Specifically, populations from areas of low water temperature and low salinity had higher skin lesion prevalence and severity [65].

Odontocetes can and do spend time in estuarine water, and bottlenose dolphins will preferentially seek out water with salinity >8 ppt [80]. A retrospective study with bottlenose dolphins revealed that epidermal lesion prevalence increased as the salinity of the water decreased, and that skin lesions were a product of low salinity and duration of exposure [42]. While there is high seasonal variability in sea surface temperature (SST) and sea surface salinity (SSS) in the inland and coastal waters inhabited by Southern Resident killer whales, data from British Columbia (Canada) light house stations indicate long-term warming and marine freshening at most stations [81]. Despite increasing long-term trends, the inland waters of the Salish Sea and outer coastal waters SSS average 29 ppt and 34 ppt, respectively [82], far higher salinity levels than those shown to initiate epidermal changes in bottlenose dolphins [41,42]. Declining salinity is not likely an underlying cause of skin disease in Southern Resident killer whales. Salinity studies with slight salinity changes in captive killer whales could help accept or reject this hypothesis.

It is unlikely that the slight ocean warming trend in the Salish Sea SST is the cause of increasing point prevalence of gray patches and gray targets in Southern Resident killer whales. Studies on captive [40] and free-ranging [65,83] bottlenose dolphins have shown that prevalence of skin disease decreases as water temperatures increase, possibly related to increased vascularization of skin permitting increased epithelial renewal and shedding. However, killer whales have a lower thermal comfort zone than bottlenose dolphins. Killer whales in human care are housed in water that is chilled to below 15.5 C and one author (HN, unpublished data) has noted that extended exposure of captive killer whales to water above 15.5 C (well above the 10 C average water temperature of the Salish Sea) was associated with skin discoloration and retention of outer epidermal layers. Conversely, Durban and Pitman [84] hypothesized that regular long-distance migration of Antarctic type B killer whales to subtropical waters might restore skin integrity by permitting epithelial shedding while maintaining thermal integrity. This suggests that longer-duration exposure to excessively cold water might precipitate some skin conditions such as the retention of un-sloughed epithelial cells and acquired diatoms, bacteria, or fungi.

Gray patches and targets could be associated with an infectious etiology such as poxvirus as in some images, they resemble tattoo skin lesions [33,85]. If this is the case, increasing trends over time could reflect impaired homeostasis or a decline in immunocompetence in Southern Resident killer whales. High levels of persistent organic pollutants (POPs) [86,87], and prey scarcity [88–90] have contributed to the decline of this population and may work independently or synergistically [91] to reduce immunocompetence in Southern Resident killer whales. Relatively speaking, POP levels are more stable and do not show seasonal or annual variation in the Southern Resident population compared to Chinook salmon (*Oncorhynchus tshawytscha*) availability, which varies considerably [90]. Thus, one could hypothesize that prevalence of gray patches and gray targets is associated with body condition and salmon abundance. Stewart et al. [91] suggest that Southern Resident killer whales have distinct interannual and pod-specific patterns of body condition fluctuation, which may be driven by pod-specific differences in foraging strategies. Data on body condition is more limited than the photographs used in our analysis, but future comparison of these datasets would be worthwhile as additional photogrammetry data are collected. If there is an immunologic component related to gray patch or gray target lesion susceptibility, we would expect more robust animals or pods that have greater foraging success to have fewer lesions. Of course, this would be expected to vary by year and pod, as we document in the point prevalence of gray patch and gray targets. Body condition has been shown to vary over much shorter time periods [92], but without data on lesion duration due to underlying gaps in animal sighting, the short-term association between skin disease and body condition might be more difficult to discern.

### Pinpoint black lesions

Pinpoint black lesions had a lower predicted point prevalence in calves and post-reproductive (older) females compared to all other stage classes (Fig 8). This was apparent but less dramatic for gray patches and gray targets as well. Without an understanding of the etiology of pinpoint black lesions, it is hard to infer why this occurs. Often, these lesions were associated with rake marks. It is possible that the act of being raked may permit entry or activation of an infectious agent such as poxvirus, as described for similar lesions in bottlenose dolphins [93] or herpesvirus as described for dusky dolphins (*Lagenorhynchus obscurus*) [94]. Alternatively, compensatory epidermal hyperplasia in response to the superficial lesion may facilitate virus replication. If either of these mechanisms are valid, the lower point prevalence in older females could be associated with older Southern Resident killer whale females having lower density of rake marks than other female age classes [56]. The rake-associated stage-class hypothesis, however, is not substantiated for calves. Female Southern Resident killer whale calves experienced significantly higher rake density than all other female age categories [56] and male calves and male juveniles exhibited over twice the rake density of subadults and three times that of adult males [56]. While Geraci et al. [93] suggest black pinpoint, grey and other lesions in dolphins are all progressions of poxvirus infection, currently, there is insufficient data to infer an etiology for these cutaneous lesions in Southern Resident killer whales.

### Cephalopod lesions

While cephalopod lesions in Southern Resident killer whales were rare, they are of interest because they suggest octopus or squid as prey for this population. Squid beaks have been found in the stomachs of both resident and transient (Bigg’s) ecotypes, though those in the transients could have come from stomach of a N. elephant seal (*Mirounga angustirostris*) that had eaten squid [10]. The squid beaks found in stomachs of two Southern Resident killer whales were from the eight-armed squid (*Gonatopsis borealis*), which reach a maximum length of 30 cm and are common in oceanic regions of the North Pacific [95]. While not quantified, lesions noted on Southern Resident killer whales were usually much longer than 30cm, so could be from other squid species such as the Humboldt squid (*Dosidicus gigas*), which is known to be expanding in range [96]. An alternate possibility is that the whales interacted with these cephalopods but did not consume them. Either way, the lesions suggest an interaction that warrants further consideration.

### Orange on gray

Epidermal diatoms are one possible etiology underlying orange on gray lesions. Diatom aggregations can create an orange film or hue on the skin of Dall’s porpoise [97], Harbor porpoise [98], Bottlenose dolphins [64,68], Guiana dolphins [9], and sperm whales [38]. The pathogenic significance of diatoms on the epithelium is likely limited. Diatoms have been recorded on the epithelium of Antarctic killer whales where they cause a more yellow, than orange hue. Hooper et al. [99] demonstrated that the extent of diatom coverage is associated with variation in the skin microbiome community, where animals with the highest diatom abundance had skin microbiomes most similar to Southern Ocean microbial communities. This suggests these animals spent more time in cold Southern Ocean waters. We hypothesize that this could be related to low epithelial turnover, which also may permit the adhesion and persistence of diatoms in Antarctic killer whales. We did not detect a seasonality in orange on gray lesions, but Southern Resident killer whales do not migrate to warmer waters in sub-tropical latitudes like some Antarctic killer whales [99], so we would not expect a seasonality to their appearance in Southern Resident killer whales if they were caused by diatoms. We saw this lesion primarily in calves. If orange on gray lesions are caused by the adherence of diatoms on the epithelium, it could be due to calves having a thicker stratum corneum layer. This epithelial layer provides insulation until the calf develops a subcutaneous blubber layer, as has been hypothesized in other cetaceans [77].

### Fetal folds

The detection of fetal folds on both sides of some animals suggests that direction of in-utero curvature may not be consistent throughout killer whale gestation. In captive killer whales, the shallow vertical fetal fold grooves in the epidermis are generally visible for approximately 30 days, though the subtle light-colored bands or striping pigmentation pattern may persist slightly longer. Unless a birth is witnessed, it can be difficult to know the exact date of parturition for wild killer whales. Knowing that the actual epidermal fetal folds or the light bands can persist for up to 55 days may improve birth date estimates.

### Calf epithelial sloughing

This condition is routinely seen in killer whales and is believed to be a normal rapid shedding of the outer epidermal stratum in neonatal calves known as postnatal ecdysis [77]. It has been reported in southern right whales from South Africa and in young belugas [77], who hypothesized that the thick stratum corneum might provide insulation for the newborn and may not be needed once the animal begins to develop a subcutaneous blubber layer. This thermoregulatory hypothesis is further supported by examples of molt in multiple age classes of bowhead whales (*Balaena mysticetus*) when they seasonally enter warmer water [100]. In captivity, killer whales can undergo multiple episodes of neonatal ecdysis (HN, unpublished data). Because weeks and months can go by between seeing wild killer whale calves, we can’t be certain if what we noted were multiple episodes of calf epithelial sloughing or just extended events. Either way, the presence of this epithelial sloughing should not be mistaken for a disease process in calves.

### Conclusions

Photographs of Southern Resident killer whales taken for identification purposes reveal six skin lesion types as well as two common skin conditions that are not considered pathologically significant. The increasing point prevalence of the highly correlated gray patches and gray targets in this population over the study period and the possible relationship between these lesions and decreasing immunocompetence is concerning. The etiology and pathogenesis of these skin lesions should be investigated.

## Supporting information

S1 Fig Total sightings by calendar day used to analyze skin lesion occurrence demonstrating concentrated effort in summer months when Southern Resident killer whales are historically in the Salish Sea. (TIFF)

S2 Fig Predicted occurrence of calf epithelial sloughing showing highest occurrence in spring and lowest in late summer. (TIFF)
