# Supplementary figures and images for: Epidemiology of skin changes in endangered Southern Resident killer whales (Orcinus orca)
Source: PLoS One. 2023 Jun 28;18(6):e0286551. doi: 10.1371/journal.pone.0286551 (PMC10306181; doi:10.1371/journal.pone.0286551)

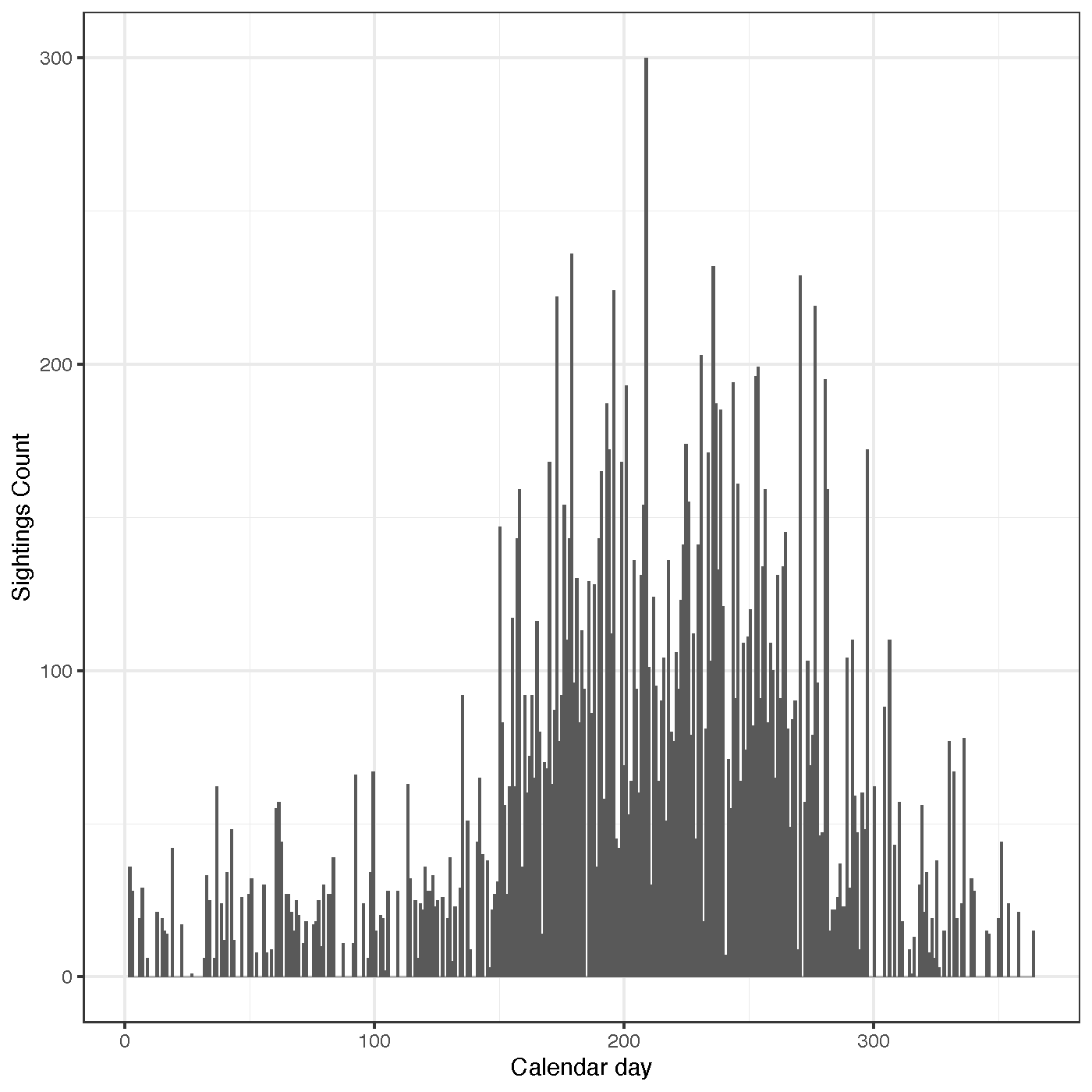

Supplement: S1 Fig — (TIFF) [file pone.0286551.s001.tiff]

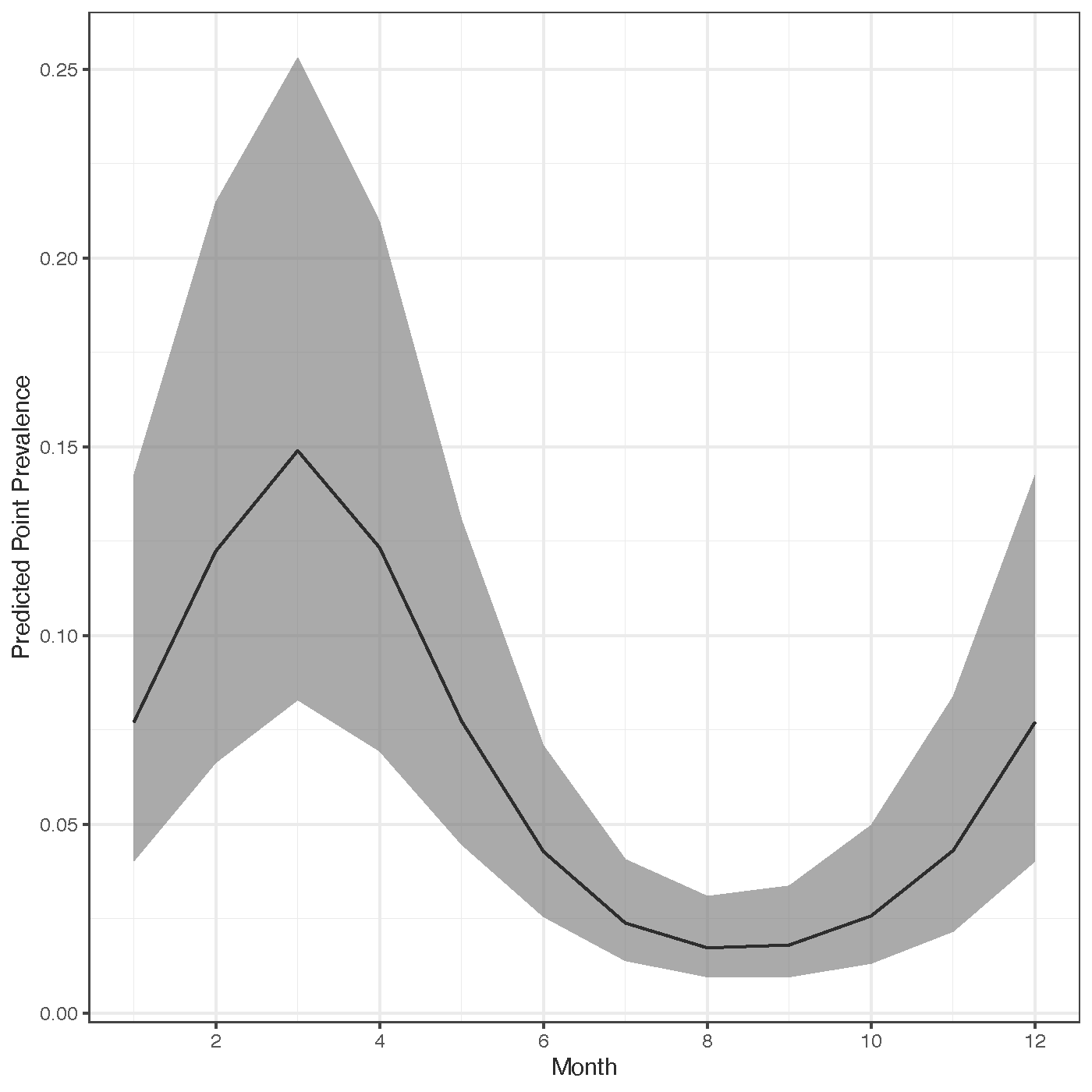

Supplement: S2 Fig — (TIFF) [file pone.0286551.s002.tiff]
